# Pre-school children single inhalation anesthetic exposure and neuro-psychological development: a prospective study and Mendelian randomization analysis

**DOI:** 10.3389/fneur.2024.1389203

**Published:** 2024-06-12

**Authors:** Jinghong Zhang, Haixia Deng, Xilu Huang, Lan Wang, Pinping Zhou, Jie Zeng, Cong Yu

**Affiliations:** ^1^Department of Anesthesiology, Stomatological Hospital of Chongqing Medical University, Chongqing, China; ^2^Chongqing Key Laboratory of Oral Diseases, Chongqing, China; ^3^Chongqing Municipal Key Laboratory of Oral Biomedical Engineering of Higher Education, Chongqing, China; ^4^People’s Hospital of Changshou, Changshou, China

**Keywords:** general anesthesia, children dental procedure, Mendelian randomization analysis, growth and development, sevoflurane, neurocognition

## Abstract

**Background:**

For children who are unable to cooperate due to severe dental anxiety (DA), dental treatment of childhood caries under Dental General Anesthesia (DGA) is a safe and high-quality treatment method. This study aims to evaluate the impact on neurocognitive functions and the growth and development of children 2 years after dental procedure based on previous research, and further establish a causal relationship between general anesthesia (GA) and changes in children’s neurocognitive functions by incorporating Mendelian Randomization (MR) analysis.

**Methods:**

Data were collected and analyzed from 340 cases of S-ECC procedures of preschool children conducted in 2019. This involved comparing the neurocognitive outcomes 2 years post-operation of preschool children receiving dental procedures under general anesthesia or local anesthesia. Physical development indicators such as height, weight, and body mass index (BMI) of children were also compared at baseline, half a year post-operation, and 2 years post-operation. We performed a Mendelian randomization analysis on the causal relationship between children’s cognitive development and general anesthesia, drawing on a large-scale meta-analysis of GWAS for anesthesia, including multiple general anesthesia datasets.

**Results:**

Outcome data were obtained for 111 children in the general anesthesia group and 121 children in the local anesthesia group. The mean FSIQ score for the general anesthesia group was 106.77 (SD 6.96), while the mean score for the local anesthesia group was 106.36 (SD 5.88). FSIQ scores were equivalent between the two groups. The incidence of malnutrition in children in the general anesthesia group was 27.93% (*p* < 0.001) before surgery and decreased to 15.32% (*p* > 0.05) after 2 years, which was not different from the general population. The IVW method suggested that the causal estimate (*p* = 0.99 > 0.05, OR = 1.04, 95% CI = 5.98 × 10^−4^–1.82 × 10^3^) was not statistically significant for disease prevalence. This indicates no genetic cause-and-effect relationship between anesthesia and childhood intelligence.

**Conclusion:**

There were no adverse outcomes in neurocognitive development in 2 years after severe early childhood caries (S-ECC) procedure under total sevoflurane-inhalation in preschool children. The malnutrition condition in children can be improved after S-ECC procedure under general anesthesia. Limited MR evidence does not support a correlation between genetic susceptibility to anesthesia and an increased risk for intelligence in children.

## 1 Introduction

Millions of children globally under the age of six suffer from severe early childhood caries (S-ECC), making it one of the most common diseases among children worldwide (approximately 50%) (1). S-ECC also remains a significant public health challenge for children in China. According to the results of the fourth national oral health epidemiological survey in Mainland China, the incidence of caries in children aged three, four, and five is 50.8, 63.6, and 71.9% respectively, most notably in the Northeast and Southwest regions of China. For children with severe dental anxiety (DA), dental treatment of childhood caries under Dental General Anesthesia (DGA) is a common practice in caries treatment. DGA is suitable for children with S-ECC who demonstrate anxious behavior or lack cognitive maturity and require lengthy dental treatments (2). However, the biggest concern for parents and pediatricians is the potential neurotoxicity of anesthesia to the developing brain, as found in animal models (3, 4). On the other hand, some renowned clinical studies did not find that general anesthesia would impair children’s neurocognitive development and concluded that anesthesia has no adverse effects on children (5, 6). Our previous research also established that, compared to local anesthesia administered while awake, the sole use of inhaled sevoflurane general anesthesia in preschool children does not have detrimental effects on neurocognitive functions 6 months post-operation. Given the potential for long-term effects, a re-evaluation of cognitive development and growth was conducted 2 years post-operation (7). Furthermore, untreated caries in a child can result in related infections that may lead to pain, eating difficulties, anxiety, sleep disorders, or psychosocial issues, eventually causing malnutrition and poor growth and development (8).

Mendelian randomization (MR) analysis, utilizing genetic variants as instrumental variables (IVs) for causal inference, has emerged as a popular method for investigating potential causal relationships between environmental exposures and diseases (9). It effectively mitigates the confounding bias and reverse causality effects seen in traditional epidemiological studies. Based on previous studies, further demonstrate the absence of a potential causal relationship between general anesthesia and postoperative changes in neurocognitive psychological function in children.

In summary, the purpose of this study is to research the impact of DGA with sevoflurane on the neurocognitive function and growth development of healthy preschool children 2 years post-procedure. In addition, we used the International Alliance data to further explore whether there are potential causal relationships in a separate two-sample MR analysis.

## 2 Methods and materials

### 2.1 Study design

We conducted a prospective, control, evaluator-blinded, equivalency trial from February to August 2019, comparing the neurocognitive outcomes of preschool children receiving dental treatment under sevoflurane-based general anesthesia or awake local anesthesia 2 years post-procedure. Follow-ups were made from February 2021 to August 2022, comparing preoperative, 6 months postoperative, and 2 years postoperative physical development indicators such as height, weight, and BMI based on the baseline. The trial was carried out at the Stomatology Hospital of Chongqing Medical University and obtained written consent from parents or guardians. The study was conducted according to the Helsinki Declaration. The trial is registered with the China Clinical Trial Registration Center (Registration No. ChiCTR1800015216) and approved by the Ethics Committee of the Stomatology Hospital of Chongqing Medical University.

The neurocognitive functions of children 2 years post-operation were assessed using the Chinese version (CN) of the Wechsler Preschool and Primary Scale of Intelligence-Fourth Edition (WPPSI-IV). The WPPSI-IV (CN) is a test scale used to assess general intelligence functions and neurocognitive development, largely used in clinical and research fields. It has high-quality standards and high clinical validity internationally. The WPPSI-IV (CN) includes 13 subtests, whose composite scores include Full-Scale IQ (FSIQ), primary indexes, and auxiliary indexes. The main outcome pre-specified per protocol for analysis was FSIQ, with secondary outcomes being selected primary indexes, including Verbal Comprehension Index (VCI), Visual Spatial Index (VSI), Working Memory Index (WMI), Fluid Reasoning Index (FRI), and Processing Speed Index (PSI).

Two hundred thirty-two children aged between 54 and 99 months who underwent DGA 2 years ago were enrolled. The initial assessment took place 6 months after the surgery. Anesthesiologists, pediatricians, and parents were aware of the group assignments, but the researchers conducting the neurofunctional assessments were not. Based on the baseline, anthropometric data were recorded. Participants were asked to wear light clothing and remove their shoes. The weight and height of the participants were measured using a weight and height ruler, accurate to 0.1 kg and 0.5 cm.

### 2.2 Participants

The inclusion criteria were children aged under 9 years and ASA I, who had completed dental caries procedures between February 1, 2019, and August 31, 2019, and had undergone an assessment using the WPPSI-IV (CN) from August 1, 2019, to March 22, 2020. Based on the Frankl behavior rating scale (FBRS), children rated as 1 (definitely negative behavior) received sevoflurane-based general anesthesia, and those with scores of 2–4 (negative, positive, and positive behavior) received conscious local anesthesia.

The exclusion criteria included any contraindications to anesthesia, previous exposure to general anesthesia, moderate to severe prematurity (gestational age not exceeding 33 weeks), extremely low birth weight (birth weight less than 1,500 grams), epilepsy, any known neural damage or developmental problems; other known diseases that might affect neural development; deafness or blindness; received any other surgery anesthesia or had history of brain trauma or other serious diseases that might affect development after undergoing dental caries procedure; and any potential reasons that might make follow-up difficult. Eligible children were recruited from the Department of Pediatric Dentistry or Anesthesiology. During the research, if a child endured brain injury or any other surgery, or failed to complete all the procedures according to the protocol, they were excluded from the study.

### 2.3 Protocol of anesthesiology

In the awake local anesthesia (LA group), local infiltration anesthesia was administered after behavior induction. For children under 4 years, 2% Lidocaine Hydrochloride Injection (5 mL: 0.1 g, Southwest Pharmaceuticals; SFDA number H50020038) was given, with a total dose not exceeding 4 mg/Kg. For children 4 years old and above, 4% Articaine Hydrochloride and Epinephrine Bitartrate Injection (1.7 mL, 68 mg, Produits Dentaires Pierre Rolland; SFDA no. H20140732) was administered, with the maximum dose not exceeding 5 mg/Kg.

The general anesthesia group (GA group) only received sevoflurane (120 mL, Shanghai Hengrui Pharmaceutical Co., Ltd.; SFDA no. H20070172) for induction and maintenance. After induction with 5 L/min oxygen and 8% sevoflurane via a face mask, the anesthesiologist chose and inserted the appropriately sized modified first-generation single-cavity laryngeal mask airway. During the maintenance phase, the anesthesiologist adjusted the sevoflurane concentration based on the vital signs and the bispectral index, most of which were 2.5–3.5% in a 2 L/min mixture of air and oxygen. The patient maintained spontaneous breathing throughout, and EtCO_2_ was usually maintained between 35 and 45 mmHg. The addition of opioid drugs, benzodiazepines, and other general anesthetics was not allowed, but local anesthetics were permissible to provide analgesia at the same dose as in the LA group. Post-anesthetic care was provided 2 h after surgery. Once the modified Aldrete score reached 10, they were allowed to leave the hospital.

### 2.4 Data collection and measurement

Demographic information, detailed obstetric information, and family structure were collected preoperatively. During the procedure, the anesthetist recorded vital signs and perioperative adverse events. Patients were followed up by phone on the day of the procedure and the first day postoperatively. Six months and 2 years postoperatively, anthropometric data were recorded, including height (Ht), weight (Wt), and body mass index (BMI). All measurements were made by physicians using standardized methods recommended by WHO. Ht was measured using a stadiometer; Wt was assessed with an electronic scale, with subjects wearing a standard minimum of clothing and no shoes. The WPPSI-IV (CN) was used to assess neurocognitive function in children from both groups. Assessments were completed 1 month after surgery and again 1 month after the 2-year mark. All children completed the evaluation alone with a qualified assessor in a one-on-one setting. Each child’s evaluation took approximately 1.5 h. Parents were asked whether they noticed any abnormalities in their child after dental treatment, and a brief physical and neurological examination of the patient was performed.

### 2.5 Statistical analysis

The statistical analysis plan presupposed age at evaluation (<7 years or ≥7 years) for subgroup analysis. The main outcome predictive variables used in the regression model included the anesthesia group, gender, gestational age at birth, birth weight, past medical history of the child, abnormal conditions during the mother’s pregnancy, the mother’s level of education, maternal age at delivery, perioperative adverse events, age at evaluation, and developmental delay after surgery. In the GA group, exposure time to sevoflurane was used as the predictive variable, and it was grouped (less than 120 min, 120–180 min, and over 180 min) to observe the impact of different anesthesia durations on the main outcomes. The analyses were done in Minitab. Using the statistical software “WHO Anthro” and “WHO AntroPlus,” the values of Ht and Wt were converted into *Z*-scores. The *Z*-score = (measured data − reference median)/reference standard deviation. This can be divided into Height-for-Age *Z*-scores (HAZ), Weight-for-Age *Z*-scores (WAZ), and BMI-for-Age (BAZ), to comprehensively evaluate the nutritional status of the child. A *Z*-score of −1.0 to −2.0 indicates mild malnutrition, −2.0 to −3.0 indicates moderate malnutrition, and below −3.0 indicates severe malnutrition. Moreover, this software used the Statistical Package for the Social Sciences (SPSS) to facilitate the analysis of research data (Chi-square-test), with *p* < 0.05 considered statistically significant.

### 2.6 Mendelian randomization analysis method

#### 2.6.1 Data source

Statistical data all comes from a large meta-analysis of Genome-Wide Association Studies (GWAS). We downloaded all the reported traits from the IEU Open GWAS Project: https://gwas.mrcieu.ac.uk/ and obtained all GWAS summary-level data related to children’s intelligence. The data regarding general anesthesia were consolidated. All of the samples were of European descent. Since the database is open access, no additional ethical approval is required. To ensure the authenticity and accuracy of causal evidence between anesthesia and intelligence risk, optimal instrumental variables (IVs) were selected. We extracted IVs significantly related to anesthesia from GWAS (*p* < 5 × 10^−6^) and removed linkage disequilibrium (LD) (*r*^2^ < 0.001, 10,000 KB). To exclude potential pleiotropic effects, we searched the PhenoScanner database^1^ for the corresponding Single Nucleotide Polymorphisms (SNPs) related to intelligence (10). We aligned the SNPs from the data sources with the same effect alleles and checked for consistency in the frequencies of the effect alleles. Ambiguous and duplicate SNPs were removed. For the screened SNPs, we employed variance (*R*^2^) and *F*-statistics to evaluate the strength of the IVs to avoid weak instrument bias (11). Using the latest robust calculation method, *F* = *R*^2^(*N* − *K* − 1)/*K*(1 − *R*^2^), *R*^2^ = 2 × (1 − EAF) × EAF×(*β*/SD)^2^, SD = SE × *N*^1/2^, where EAF is the effect allele frequency, *β* is the estimated effect, *N* is the GWAS sample size, SE is the standard error of the estimated effect. *R*^2^ refers to the cumulative variance explained by the selected SNP during exposure, and *K* is the number of SNPs used for the final analysis. If the *F* statistic of the IV in the IV strength test is more than 10, it is considered that the correlation between the IV and the exposure is strong enough, and the MR analysis result can avoid being affected by weak instrument bias.

#### 2.6.2 Study design

**Figure 1:**
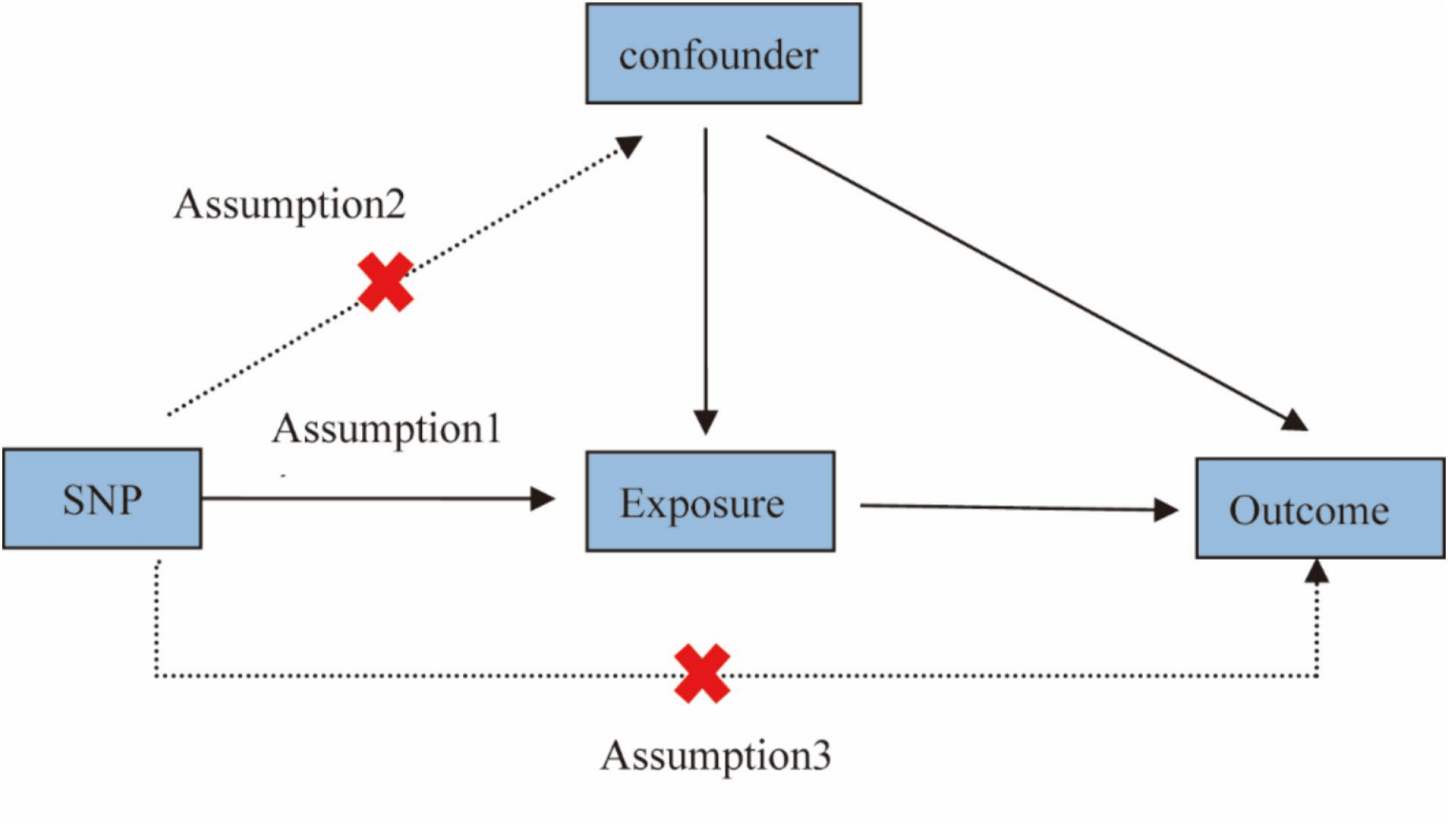
Study design for Mendelian randomization studies. Mendelian randomization is based on three assumptions: (1) Instrumental variables are closely related to exposure; (2) Instrumental variables are not associated with any confounding factors; and (3) Instrumental variables affect results only by exposure and not by other means.

We conducted a Mendelian Randomization (MR) analysis to study the causal relationship between anesthesia and intelligence. An overview of the study design is shown in Figure 1. For traits that include multiple IVs, we used Inverse Variance Weighted (IVW) as the primary method, supplemented by WeightedMedian, Weightedmode, Maximum likelihood (12), MR-Egger regression, Inverse Variance Weighted (Fixed Effect). The Cochran’s *Q* statistic (MR-IVW) and the Rucker’s *Q* statistic (MR Egger) were employed to assess the presence of heterogeneity in the MR analysis, with *p* > 0.05 indicating no evidence of heterogeneity. We applied MR-Egger regression testing to monitor potential horizontal pleiotropic effects (13–15). If *p* > 0.05, it indicates that there is no evidence of horizontal pleiotropy in the IV. We used the MR-PRESSO to detect whether there were outliers in our MR analysis. To improve the robustness of the results, this study introduced a variety of sensitivity analysis methods and conducted several sensitivity analyses. A leave-one-out analysis was conducted to determine whether the causal signal is driven by a single SNP. The statistical analyses were performed using the “Two Sample MR” and “MRPRESSO” package in R software.

## 3 Results

### 3.1 General data of follow-up

**Figure 2:**
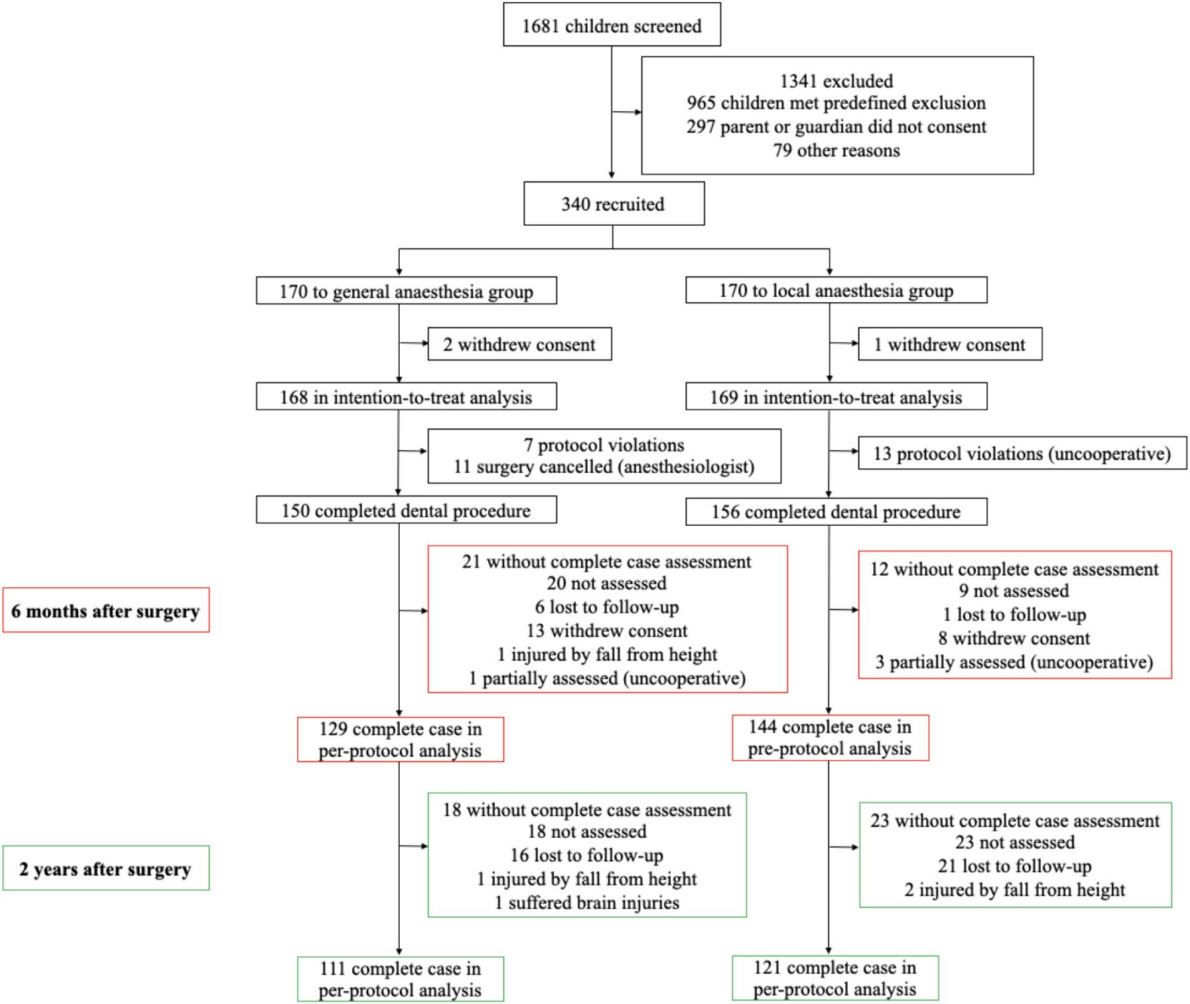
Research flow chart.

From February 1, 2019, to August 31, 2019, 1,681 children were screened for eligibility, and 340 patients were recruited. After the withdrawal of consent by parents on three occasions (pre-surgery), the intention-to-treat analysis included 168 children in the GA group and 169 in the LA group. After excluding 31 participants who either had their surgeries canceled or violated protocols, 150 patients in the GA group and 156 in the LA group completed their operations. The first follow-up was conducted from August 1, 2019, to March 22, 2020. Twenty eight families were lost to follow-up or withdrew consent, and one child was injured in a fall from a height. In the per-protocol analysis, the WPPSI-IV (CN) was completed by 129 individuals in the GA group and by 144 in the LA group. The second follow-up took place from February 1, 2021, to August 31, 2022. A total of 37 families were lost to follow-up, three children were injured by fall from a height, and one child received head injuries. In the per-protocol analysis, the WPPSI-IV (CN) was completed by 111 individuals in the GA group and by 121 in the LA group (Figure 2).

### 3.2 Baseline demographic data and non-scale measures

Post-surgery baseline demographic data and anesthesia details are presented in Table 1. The GA group included 63 boys (49%), and the LA group had 77 boys (53%). The age at surgery ranged from 2.5 to 6.5 years. Among all participants, 9 mothers had slight anomalies during pregnancy, and 12 children had a past medical history. The median time of sevoflurane usage was 130 min (ranging from 110 to 160 min). The only perioperative adverse reaction in the general anesthesia group was related to respiratory system complications (two mild cases of laryngospasm), and there was one case of local anesthesia adverse reaction in the LA group.

**Table 1:** Post-surgery base demographic data and anesthesia details.

	GA group	LA group	*p* value^a^
Baseline demographics			
Sex, M/F	63/66	77/67	0.444
Age at surgery			
<4 years old	36	22	
≥4 years old	93	122	
Weight, kg; median (IQR)	17.50 (15.80–18.70)	18.35 (16.50–20.20)	<0.001
Height, cm; median (IQR)	106 (99.50–110.00)	106 (101.00–115.00)	0.003
Past medical history			
Non-recurrent febrile convulsion	2 (2%)	3 (2%)	1.000
Thalassemia without a history of blood transfusion	3 (2%)	0	0.104
Favism	0	1 (<1%)	1.000
Patent ductus arteriosus without treatment	2 (2%)	1 (<1%)	0.604
Pregnancy and birth details			
Pregnancy (born < 37 weeks’ gestation)	8 (6%)	5 (3%)	0.290
Birthweight, kg	3.30 (0.53)	3.29 (0.46)	0.909
One of a multiple pregnancy	2 (2%)	1 (<1%)	0.604
Abnormal pregnancy examination of mother	4 (3%)	5 (3%)	1.000
Delivery mode			
Cephalic vaginal	62 (48%)	73 (51%)	0.664
Cesarean section with intravertebral anesthesia	67 (52%)	71 (49%)	
Family demographics			
Maternal age at birth more than 30 years	33 (26%)	45 (31%)	0.302
Maternal education			
Junior high school	7 (5%)	5 (3%)	0.486
Senior high school	31 (24%)	27 (19%)	
Bachelor degree	82 (64%)	108 (75%)	
Master degree or higher	9 (7%)	4 (3%)	
Number of children in the family			
1	83 (64%)	92 (64%)	0.909
2	45 (35%)	50 (35%)	
3	1 (<1%)	2 (1%)	
Birth order			
1	108 (84%)	114 (79%)	0.324
2	21 (16%)	29 (20%)	
3	0	1 (<1%)	
Anesthesia details			
Duration of surgery, min; median (IQR)	115 (95–140)	NA	
Duration of use of sevoflurane, min; median (IQR)	130 (110–160)	NA	
Adverse events related to the cardiovascular system	0	1 (<1%)^b^	1.000
Adverse events related to the respiratory system	2 (2%)^c^	0	0.222
Apparent hypoxia^d^	0	0	

Data are presented as *n*, means (standard deviations), or median (IQR).

a Pearson’s chi-squared test, Fisher’s exact test, Rank sum test, or Two independent samples *t*-test.

b One patient had an adverse reaction to local anesthetic with increased heart rate (recovered after treatment).

c Two children had mild laryngospasm without evident hypoxia during anesthesia recovery.

d Apparent hypoxia defined as oxygen saturation < 90%.

The non-scale measurement results 2 years post-surgery are presented in Table 2. There were 66 children under 7 years old in the GA group and 64 in the LA group. Post-operation, no child was diagnosed with febrile convulsions, epileptic seizures, or cerebral palsy, nor did any fail to complete the WPPSI-IV (CN) due to developmental issues or behavioral disorders. All children had normal neurological examinations.

**Table 2:** Non-scale measurement at 2 years after DG.

	GA group (*n* = 111)	LA group (*n* = 121)	*p* vaule^a^
Assessment details			
Age at follow-up assessment			
<7 years old	66	64	
≥7 years old	45	57	
Weight, kg; median (IQR)	22.00 (16.90–29.60)	23.80 (17.60–28.50)	0.030
Height, cm; median (IQR)	120.00 (105.00–136.00)	122.00 (108.00–132.00)	0.423
Abnormal neurological examinations	0	0	
Events after caries treatment			
Febrile convulsion or epileptic seizure	0	0	
Cerebral palsy	0	0	
Developmental delay	0	0	
Hearing or vision impairment	0	0	
Language, behavior or psychomotor disorder	0	0	
Intervention for neurodevelopmental problem	0	0	

Data are presented as n or median (IQR).

^a^ Pearson’s chi-squared test or Two independent samples *t*-test.

**Figure 3:**
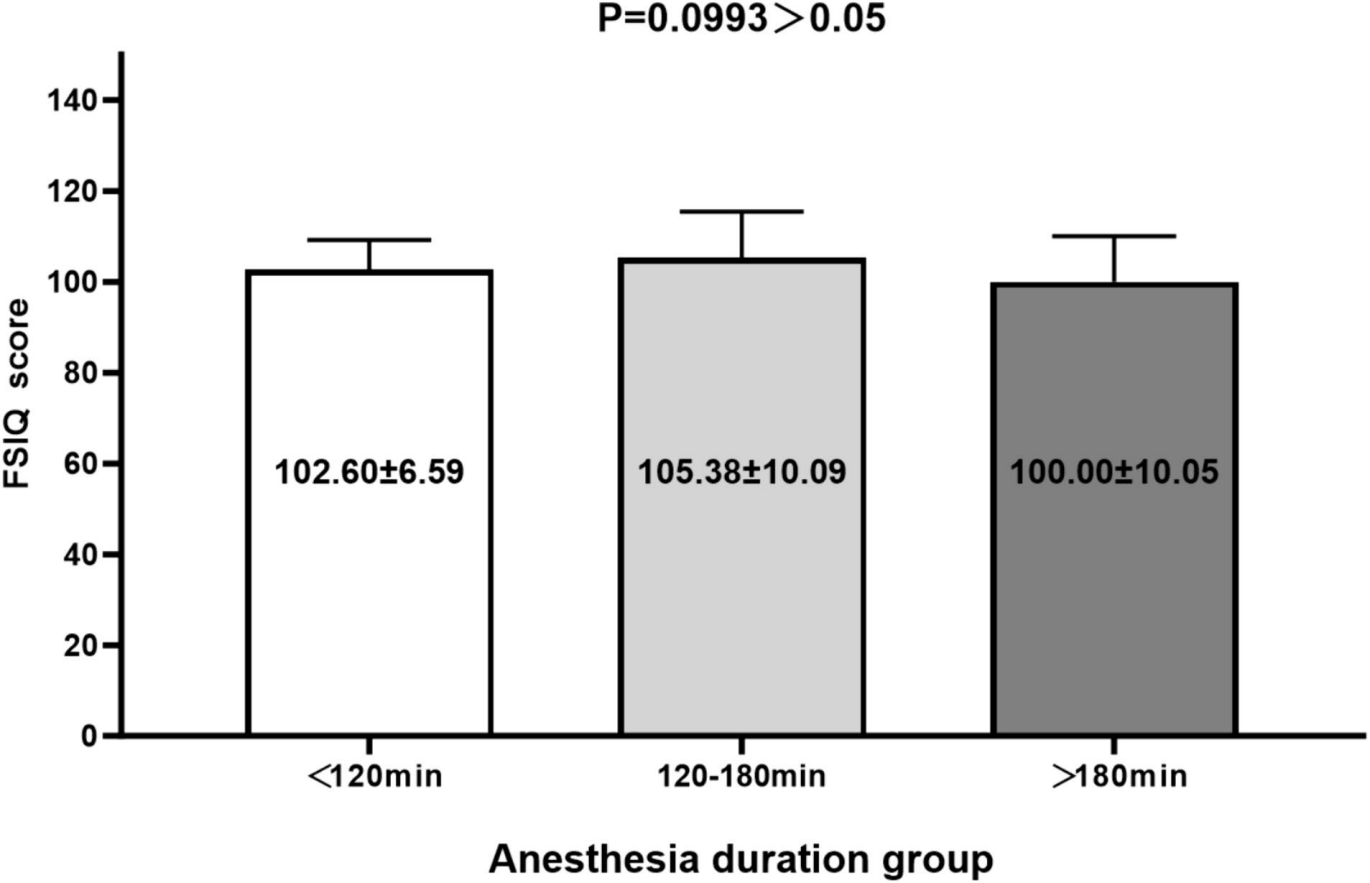
Comparison of the FSIQ scores between groups of anesthesia duration.

There was no significant difference in FSIQ score between groups (less than 120 min, 120–180 min, and more than 180 min) of different anesthesia durations (*p* = 0.0993 > 0.05) (as shown in Figure 3).

### 3.3 General results of measurements

The FSIQ scores and the main indicators of the WPPSI-IV (CN) (VCI, VSI, FRI, WMI, and PSI) for the two groups of patients are presented in Table 3. In terms of FSIQ scores, the average for the GA group was 106.77 (SD 6.96), and for the LA group, the average was 106.36 (SD 5.88). Evidence suggested equivalence in the mean values between the two groups (The average difference between local and general anesthesia being −0.41, 95% CI –1.80 to 1.00, the average difference is within this range). There was also evidence that the VCI, VSI, FRI, WMI, PSI, and PRI scores were comparable between the two groups. In all analyses, the upper and lower limits of the 95% CI were well within the pre-set range of equivalence (5 times).

**Table 3:** Comparison of WPPSI-IV (CN) scores between groups.

Composite score	GA group (*n* = 111)	LA group (*n* = 121)	Difference in LA-GA	95% CI for difference in LA-GA
FSIQ	106.77 (6.96)	106.36 (5.88)	−0.41	(−1.80, 1.00)
VCI	105.26 (8.18)	104.58 (8.26)	−0.68	(−2.47, 1.10)
VSI	105.47 (8.35)	105.83 (7.70)	0.36	(−2.69, 1.98)
FRI	106.26 (9.13)	103.70 (8.34)	−2.56	(−5.10, 0)
WMI	103.97 (8.83)	103.57 (8.91)	−0.40	(−2.33, 1.52)
PSI	103.79 (9.11)	104.00 (7.85)	0.21	(−1.64, 2.06)
PRI	105.80 (7.74)	105.16 (8.35)	−0.64	(−3.30, 2.01)

Data are means (SD).

WPPSI-IV (CN), Wechsler Preschool and Primary Scale of Intelligence-Fourth Edition (Chinese version); FSIQ, Full Scale IQ; VCI, Verbal Comprehension Index; VSI, Visual Spatial Index; FRI, Fluid Reasoning Index; WMI, Working Memory Index; PSI, Processing Speed Index.

### 3.4 Subgroup analysis and predictive variable analysis of scale measurements

In the grouped analysis, the FSIQ scores for the age group under 7 years old were comparable between GA group and LA group in Table 4. The scores for the age group over 7 years were also similar. There was no significant difference between the two age groups in either GA group or LA group.

**Table 4:** Comparison of WPPSI (CN) scores in age subgroups.

Composite score	<7 yrs GA group (*n* = 66)	<7 yrs LA group (*n* = 64)	Difference (<7 yrs) LA-GA (95% CI)	≥7 yrs GA group (*n* = 45)	≥7 yrs LA group (*n* = 57)	Difference (≥7 yrs) LA-GA (95% CI)
FSIQ	106.86 (7.77)	105.17 (5.37)	−1.69 (−3.63, 0.25)	106.62 (5.65)	107.70 (6.18)	1.08 (−0.87, 3.03)
VCI	106.39 (8.24)	104.81 (8.18)	−1.58 (−3.97, 0.81)	103.60 (7.88)	104.32 (8.41)	0.72 (−1.97, 3.41)
VSI	105.47 (8.35)	105.83 (7.70)	0.36 (−1.98, 2.69)	NA	NA	NA
FRI	106.26 (9.13)	103.70 (8.34)	−2.56 (−5.10, 0)	NA	NA	NA
WMI	102.05 (9.05)	102.58 (8.27)	0.53 (−1.99, 3.05)	106.80 (7.74)	104.68 (9.53)	−2.12 (−4.96, 0.72)
PSI	104.36 (8.81)	104.88 (8.70)	0.52 (−2.03, 3.06)	102.96 (9.58)	103.02 (6.71)	0.06 (−2.74, 2.86)
PRI	NA	NA	NA	105.80 (7.74)	105.16 (8.35)	−0.64 (−3.30, 2.01)

Data are means (SD).

WPPSI-IV (CN), Wechsler Preschool and Primary Scale of Intelligence-Fourth Edition (Chinese version); FSIQ, Full Scale IQ; VCI, Verbal Comprehension Index; VSI, Visual Spatial Index; FRI, Fluid Reasoning Index; WMI, Working Memory Index; PSI, Processing Speed Index.

*Z*-fraction can be used to compare an individual’s Wt, Ht or BMI, adjusted for age and gender relative to a reference population. *Z*-fraction of −1.0 to −2.0 suggest mild malnutrition, −2.0 to −3.0 indicate moderate malnutrition, and scores below −3.0 signify severe malnutrition. The values of height and weight are converted into *Z*-scores using the statistical software “WHO Anthro” and “WHO AntroPlus,” and then child nutrition is assessed through these *Z*-scores. According to the study, the prevalence of malnutrition in children in the general population was 10.93% (2022/18503) (16). The data obtained in this survey was compared with the figures in Table 5. Before treatment, 31 of 111 children in the general anesthesia group were malnourished (27.93%), a significantly higher proportion than the proportion of malnourished children in the general population (*p* < 0.05). After DGA, the proportion of malnourished children gradually decreased. After 6 months of DGA, the proportion of malnourished children was lower but still higher than the prevalence of malnourishment in children in the general population (*p* = 0.037 < 0.05). After 2 years of DGA, the proportion of malnourished children was no longer significantly different from the general population (*p* = 0.140 > 0.05). In the LA group, the proportion of malnourished children before and after treatment was not significantly different from the general population (*p* > 0.05).

**Table 5:** Comparison of prevalence of malnutrition pre- and post-dental procedure.

	Time of procedure	Number of malnutrition	Incidence of malnutrition	*p* vaule^a^
GA Group (*n* = 111)	Pre-dental procedure	31	27.93%	<0.001^b^
	6-months post-dental procedure	19	17.12%	0.037^b^
	24-months post-dental procedure	17	15.32%	0.140
LA Group (*n* = 121)	Pre-dental procedure	12	9.92%	0.722
	6-months post-dental procedure	9	7.44%	0.22
	24-months post-dental procedure	7	5.79%	0.07

a Pearson’s chi-squared test.

b The significance level < 0.05.

### 3.5 Mendelian analysis results

#### 3.5.1 Correlation analysis of MR

We selected ieu-a-16 from the SSGAC database for MR (Mendelian Randomization) analysis, which is shown in Table 6. The Inverse variance weighted (IVW), Maximum likelihood, Weighted mode, Weighted median and MR-Egger (17) analysis results showed that anesthesia had no genetic causal relationship with childhood intelligence (*p* > 0.05).

**Table 6:** Mendelian randomization estimates of anesthetics on the risk for childhood intelligence.

	SNPs (*n*)	OR	95%CI	*p*-value
MR Egger	16	0.02	4.91 × 10^−12^–7.53 × 10^7^	0.73
Maximum likelihood	16	1.05	5.32 × 10^−4^–2.06 × 10^3^	0.99
Weighted mode	16	1.27	5.70 × 10^−7^–2.84 × 10^6^	0.97
Weighted median	16	0.73	2.37 × 10^−5^–2.24 × 10^4^	0.95
IVW	16	1.04	5.98 × 10^−4^–1.82 × 10^3^	0.99

SNPs, Single nucleotide polymorphisms; OR, Odds ratio; CI, Confidence interval; IVW, inverse-variance weighted; IVW (fixed), Inverse variance weighted (fixed effects); Weighted median.

**Figure 4:**
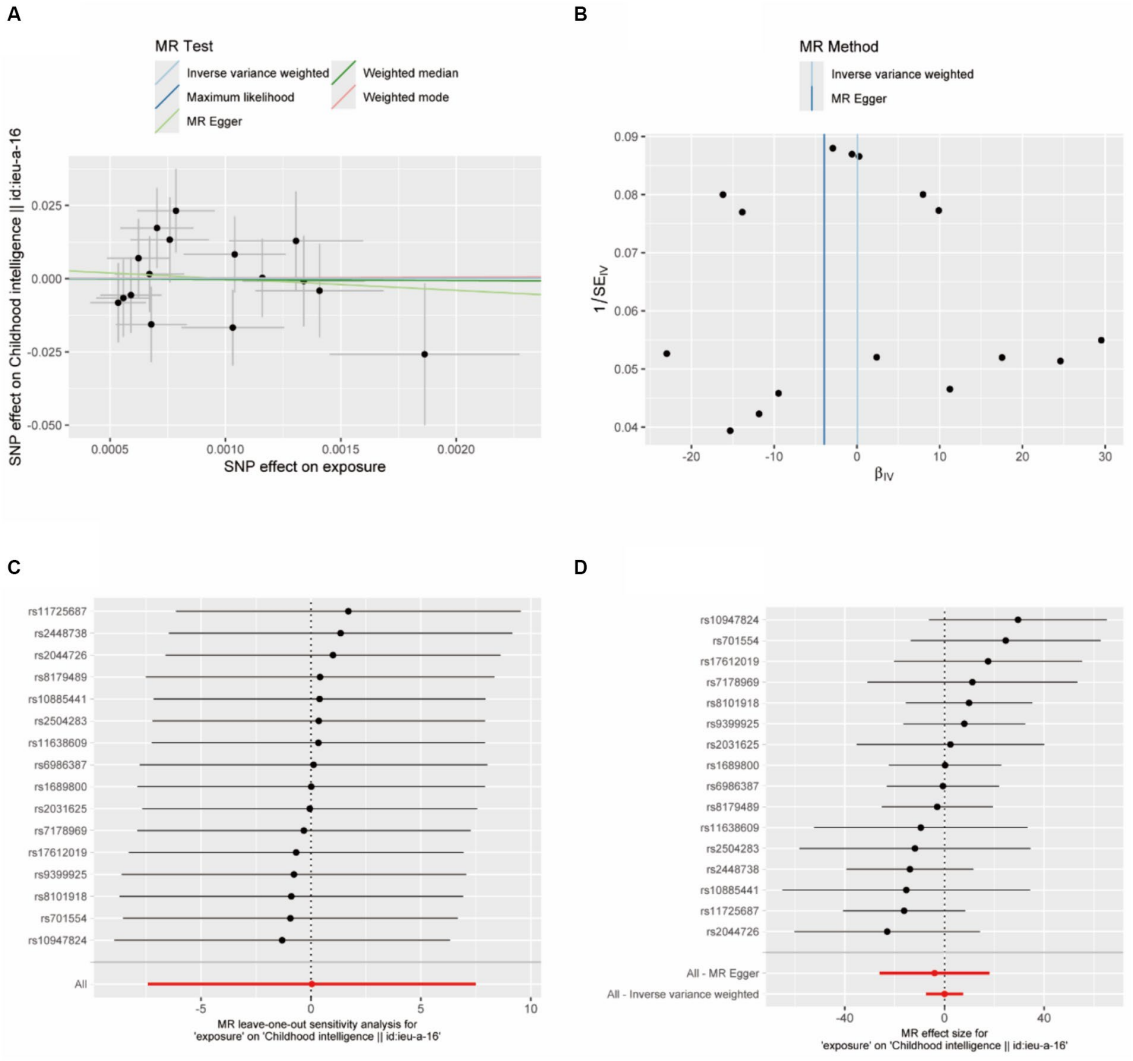
**(A)** Scatter plot: Each point in this plot represents an IVs, and the lines at each point reflect the 95% confidence interval. The horizontal coordinate is the effect of SNP on exposure (anesthesia), the vertical coordinate is the effect of SNP on outcome (child intelligence), and the colored lines represent the MR Fitting results; **(B)** funnel diagram; **(C)** forest diagram, MR effect size; **(D)** MR leave-one-out sensitivity analysis.

The horizontal solid lines in Figure 4 of the forest plot represent the estimates derived from individual SNPs using the Wald ratio method. Solid lines positioned to the left of 0 indicate a reduced risk based on the SNP’s estimate, while those to the right suggest an increased risk. Lines crossing 0 signify results that are not statistically significant. There may be issues with the results of examining the outcomes of a single SNP, as more reliable results are obtained when all results are considered collectively.

#### 3.5.2 Sensitivity analysis of MR

In the heterogeneity test, the *p*-value of Cochran’s *Q* statistic is greater than 0.05, indicating no heterogeneity between SNPs (Table 7). Therefore, in this MR analysis, we use the fixed-effects Inverse Variance Weighting (IVW) method as the primary analysis method. Besides, the MR-regression intercept point J entirely to the left of Egger indicates limited evidence of pleiotropy in any anesthetic IVs for child intelligence (Figure 4B). Regardless of which SNP is removed, it would not fundamentally affect the results (all lines are to the left of 0), meaning that this MR result is robust. The causal association between anesthesia and intelligence risk is not driven by a single SNP (Figure 4D). The global test of MR-PRESSO showed that the MR analysis had no horizontal pleiotropy (*p* > 0.05).

**Table 7:** Pleiotropy and heterogeneity test of the anesthetics IVs from childhood intelligence GWAS.

Outcome	Pleiotropy test MR-Egger			Heterogeneity test MR-Egger			Inverse-variance weighted			MR-PRESSO global test	
	Intercept	SE	*p*	*Q*	*Q* df	*Q p*val	*Q*	*Q* df	*Q* pval	Outlier	*p*
Childhood intelligence	0.0039	0.01	0.71	11.33	14	0.66	11.48	15	0.717	None	0.725

df, degree of freedom; MR, Mendelian randomization; *Q*, heterogeneity statistic Q; MR-PRESSO, MR-Pleiotropy Residual Sum and Outlier method. MR-Egger regression shows that there is no unbalanced horizontal pleiotropy (MR-Egger Intercept = 0.0039; *p* = 0.71). No outliers were detected by radial MR Method.

## 4 Discussion

The debate regarding the impacts of general anesthetic agents on Central Nervous System (CNS) development in children has been ongoing for nearly 20 years. Currently, it is generally agreed that temporary or single early exposure to anesthesia in children is not associated with defects in a range of neurodevelopmental outcomes (including broad intelligence measures). To provide clearer and more potent clinical research evidence, we continued a comparative study initiated in 2019 evaluating the neurocognitive development 2 years post-caries treatment in children who underwent either general or local anesthesia. Specifically, we examined the equivalence of evidence between general and local anesthesia in terms of FSIQ in the WPPSI-IV (CN) measured 2 years after the procedure. VCI, VSI, FRI, WMI, PSI, and PRI also showed equivalence. Children who received long-term DGA solely with sevoflurane showed no adverse neurocognitive development changes when compared to the LA group at 2 years post-operatively, the same conclusion as our previous survey (7).

Few randomized controlled trials or prospective studies have been conducted in the field of safety of general anesthetic agents in children, with most studies being observational. However, observational studies may contain many potential confounders, and the data from such research is often collected for other purposes. In terms of experimental design, some articles suggest that studies should focus on consistent surgical procedures involving one type of surgery, anesthetic agents, and less harmful surgeries to eliminate potential confounding factors (18). This was considered in our experimental design. Aside from the causal effects of anesthetic exposure, various factors such as the disease, surgery, and recovery process can lead to postoperative neuropsychological development adverse outcomes. These factors include perisurgical physiological disorders, surgery-related inflammation, psychological stress, and physiological disorders caused by underlying diseases that necessitate surgery including hypoxia, hypocapnia, hypercapnia, and impacts on cerebral tissue perfusion and oxygenation. We designed a dental caries treatment that quickly recovers postoperatively, employs a single inhaled anesthetic, effectively circumvents the aforementioned influencing factors, and avoids the impact of multiple drug use on the results, making it optimized for long-term follow-ups. Three authoritative studies, namely a randomized controlled trial (RCT) comparing sevoflurane anesthesia (GAS) with spinal anesthesia; the Mayo Anesthesia Safety in Kids (MASK) study related to other anesthetics; and the Pediatric Anesthesia and Neurodevelopmental Assessment (PANDA) study, all concluded that anesthetic exposure is unrelated to pediatric neurodevelopment. This provides clinical evidence of the safety of general anesthesia on pediatric neurodevelopment (6, 19, 20). However, identifying which children are most susceptible remains a challenge. Many diseases require repeated and prolonged exposure to anesthesia, and quantifying this increased risk is challenging. In addition to medical factors, family and social factors also play significant roles, such as socioeconomic status and maternal education level, making it unclear as to what the critical vulnerability window for brain development is (21). A recent systematic review and meta-analysis of 31 studies found a statistically significant correlation between receiving general anesthesia during childhood and increased incidences of behavioral problems, diagnoses of neurodevelopmental disorders, deficits in executive function, non-verbal reasoning, motor function, and language, development, and learning (to a minor extent). Differences in cognitive scores, while statistically significant, were the least associated (22). This research provides a valuable conclusion that the correlation between anesthetic exposure and neurodevelopment deficits may be different based on the specific neurodevelopmental domain assessed.

The dispute between anesthetic drugs and cognitive development stems from the contradictory results between laboratory evidence and clinical practice. Basic research has replicated various morphological and functional changes caused by general anesthetic drugs at different stages of brain development in models from rodents to non-human primates, providing methods and ideas for the adverse outcomes of the nervous system caused by different anesthetic drugs, anesthetic duration, and multiple anesthetic exposures (4, 23, 24). Meanwhile, the molecular mechanisms of the effects of general anesthesia on brain development are widely explored, including mechanisms such as induced cell apoptosis via intrinsic and extrinsic pathways (25), obstructed connections with adjacent neurons, axonal myelin formation disorders, and CNS inflammation responses caused by anesthetic drugs (26). However, basic research is still unable to provide convincing evidence for different drug effects and long-term follow-up results. Therefore, in addition to using random control experimental designs, we also used the method of Mendelian randomization analysis to detect and quantify the impact of anesthetic exposure on neuro-psychological development, excluding the interference of confounding factors, to further explain their causal relationship. Currently, limited MR evidence does not support the correlation of genetic susceptibility to anesthesia with an increased risk of intelligence in children.

Using a two-sample Mendelian randomization (MR) analysis, we can effectively avoid the confounding bias of traditional epidemiological research. We extracted 78 SNPs from the Integrated anesthesia data set dataset. After excluding 62 SNPs that were related to child intelligence or that were duplicates or unclear, we obtained 16 SNPs. We used various MR methods to verify these 16 SNPs and obtained almost bias-free results—the evidence of no causal relationship between anesthesia and child intelligence does indeed exist. However, there were relatively few SNPs used as instrumental variables for the analysis. Secondly, both the summarized data sets for MR exposure and outcome were from Europeans. We cannot rule out the possibility of accidental findings due to specific lineage genetic variations. The causal relationship between the two still needs to be verified in MR analysis using genetic data from a Chinese cohort or more data sources.

Research indicates that dental caries can directly affect children’s nutritional status and growth and development (27). Evidence supporting an inverse relationship between dental caries and BMI comes from studies in developing countries. Severe caries can likely impair a child’s ability to eat, leading to malnutrition (28). BMI is an important indicator of nutritional status. In our study, we grouped the children based on the severity of their dental caries to suggest whether the treatment room should adopt general or local anesthesia for treatment. For children suffering severe caries and longer surgical time, we recommend treatment under general anesthesia. Children with more severe dental caries often fall behind their peers in growth and development. The malnutrition rate among children in the GA group was higher than the general population before treatment. In this study, 27.93% of children had poor nutritional status before the procedure, after DGA, the children improved their eating efficiency and formed a healthy eating pattern. In addition, many parents accepted the dentist’s advice to correct poor dietary habits. Therefore, 2 years after treatment, the incidence of malnutrition in the children decreased compared to before treatment and was no different from the general population. We found that the incidence of malnutrition among the children 2 years after treatment was no different from the general population, indicating that prompt and comprehensive treatment of dental caries can help to improve the children’s nutritional status.

## 5 Conclusion

In relatively healthy preschool children undergoing treatment for S-ECC under general anesthesia, no evidence was found that the use of sevoflurane-based general anesthesia alone would lead to adverse neurocognitive outcomes (including language, reasoning, memory, and visual-motor) after 2 years survey. The MR analysis also found no evidence that anesthesia affects post-surgical intelligence development in children. Although these are not conclusions, these findings should reassure pediatricians and parents not to consider postponing surgery because of brain development concerns and medical risks. Regardless of the anesthesia method, timely treatment of dental caries can significantly improve malnutrition in affected children. However, the following limitations also deserve attention. We acknowledge the inevitable the loss-of-follow-up ratio, especially in the GA group. Next as the Mendelian randomization analysis was conducted exclusively on individuals of European descent, we were unable to perform further validation in our study. We thank all participants and researchers who participated in this MR study. The IEU Open GWAS project and the European Bioinformatics Institute GWAS Catalog provided the summary data for the analysis.

## Data availability statement

The original contributions presented in the study are included in the article/supplementary material, further inquiries can be directed to the corresponding author.

## Ethics statement

The studies involving humans were approved by China Clinical Trial Registration Center (Registration No. ChiCTR1800015216) and the Ethics Committee of the Stomatology Hospital of Chongqing Medical University. The studies were conducted in accordance with the local legislation and institutional requirements. Written informed consent for participation in this study was provided by the participants’ legal guardians/next of kin.

## Author contributions

JZh: Conceptualization, Data curation, Formal analysis, Validation, Writing – original draft. HD: Investigation, Writing – review & editing. XH: Conceptualization, Data curation, Formal analysis, Validation, Writing – original draft. LW: Formal analysis, Writing – review & editing. PZ: Investigation, Writing – review & editing. JZe: Investigation, Writing – review & editing. CY: Conceptualization, Writing – review & editing.

## Footnotes

^1^www.phenoscanner.medschl.cam.ac.uk
